# NPInter v3.0: an upgraded database of noncoding RNA-associated interactions

**DOI:** 10.1093/database/baw057

**Published:** 2016-04-16

**Authors:** Yajing Hao, Wei Wu, Hui Li, Jiao Yuan, Jianjun Luo, Yi Zhao, Runsheng Chen

**Affiliations:** ^1^Key Laboratory of RNA Biology; ^2^Beijing Key Laboratory of Noncoding RNA, Institute of Biophysics, Chinese Academy of Sciences, Beijing, 100101, China; ^3^University of Chinese Academy of Sciences, Beijing, 100049, China; ^4^Bioinformatics Research Group, Key Laboratory of Intelligent Information Processing, Advanced Computing Research Center, Institute of Computing Technology, Chinese Academy of Sciences, Beijing, 100190, China

## Abstract

Despite the fact that a large quantity of noncoding RNAs (ncRNAs) have been identified, their functions remain unclear. To enable researchers to have a better understanding of ncRNAs’ functions, we updated the NPInter database to version 3.0, which contains experimentally verified interactions between ncRNAs (excluding tRNAs and rRNAs), especially long noncoding RNAs (lncRNAs) and other biomolecules (proteins, mRNAs, miRNAs and genomic DNAs). In NPInter v3.0, interactions pertaining to ncRNAs are not only manually curated from scientific literature but also curated from high-throughput technologies. In addition, we also curated lncRNA–miRNA interactions from *in silico* predictions supported by AGO CLIP-seq data. When compared with NPInter v2.0, the interactions are more informative (with additional information on tissues or cell lines, binding sites, conservation, co-expression values and other features) and more organized (with divisions on data sets by data sources, tissues or cell lines, experiments and other criteria). NPInter v3.0 expands the data set to 491,416 interactions in 188 tissues (or cell lines) from 68 kinds of experimental technologies. NPInter v3.0 also improves the user interface and adds new web services, including a local UCSC Genome Browser to visualize binding sites. Additionally, NPInter v3.0 defined a high-confidence set of interactions and predicted the functions of lncRNAs in human and mouse based on the interactions curated in the database. NPInter v3.0 is available at http://www.bioinfo.org/NPInter/.

**Database URL**: http://www.bioinfo.org/NPInter/

## Introduction

Over the past decade, numerous noncoding RNAs (ncRNAs) have been identified in human (1), mouse (2) and other organisms (3– 5) due to the advances in high-throughput sequencing (6). Emerging evidence has suggested that, except for the well-recognized ncRNAs such as rRNAs (7), tRNAs (8) and small nuclear RNAs (9), other regulatory ncRNAs, such as miRNAs (10), siRNAs (11), piRNAs (12), and the recently rapidly expanding class of long noncoding RNAs (lncRNAs) play key roles in a range of biological processes, including genomic imprinting, disease metastasis, cell pluripotency and differentiation, and many others (13– 15).

ncRNAs are known to function by interfacing with diverse classes of biomolecules. For example, miRNAs associate with Argonaute proteins to form miRNA-induced silencing complexes to regulate the expression of mRNA targets (16). The lncRNA, Xist, physically interacts with different factors to initiate and maintain the processes of X chromosome silencing (17). Therefore, identifying a more complete spectrum of ncRNAs interacting partners will significantly deepen the understanding of how ncRNAs modulate biological processes. Towards this end, many recent molecular experimental approaches combined with high-throughput sequencing or mass spectrometry were carried out to identify these interactions, such as protein-centric approaches, crosslinking and immunoprecipitation followed by deep sequencing (CLIP-seq) (18), RNA-centric approaches, Chromatin isolation by RNA purification followed by high-throughput sequencing (ChIRP-seq) (19), and others (20– 22).

With the widespread application of these new high-throughput technologies and the explosive data accumulation of interactions between RNA and other biomolecules, we initiated a project to build a data repository and platform for cataloguing their interactions (NPInter (23)), and successfully updated to version 2 (24) which expanded the data collection and introduced tools for data visualization. However, the large amount of new research, particularly studies on CLIP-seq, has largely overwhelmed the collection of ncRNAs’ interactions in NPInter v2.0. Thus, NPInter have been upgraded to version 3.0 to collect substantially more interactions from the literature, high-throughput sequencing, and *in silico* predictions supported by high-throughput sequencing data. In addition, ncRNAs were given accession IDs from NONCODE (25– 28), RefSeq (29), Ensembl (30), and miRBase (31) while protein-coding molecules were assigned from UniProt (32), UniGene and RefSeq. Binding site information was appended to interactions discovered by CLIP-seq with conservation scores. Gene expression correlation scores were also added to the descriptions of the interactions by co-expression analysis. Owing to the fact that the number of interactions had become quite large, NPInter v3.0 also provided a high-confidence set of interactions and reorganized interactions according to different aspects such as the source of the data, tissues or cell lines, experiments and other factors. Moreover, we predicted the functions of lncRNAs in human and mouse based on the interactions curated in the database. Furthermore, we designed a new website and integrated a Genome Browser service, which greatly improved the interface and user experience. All data are available on the download page.

**Figure 1:**
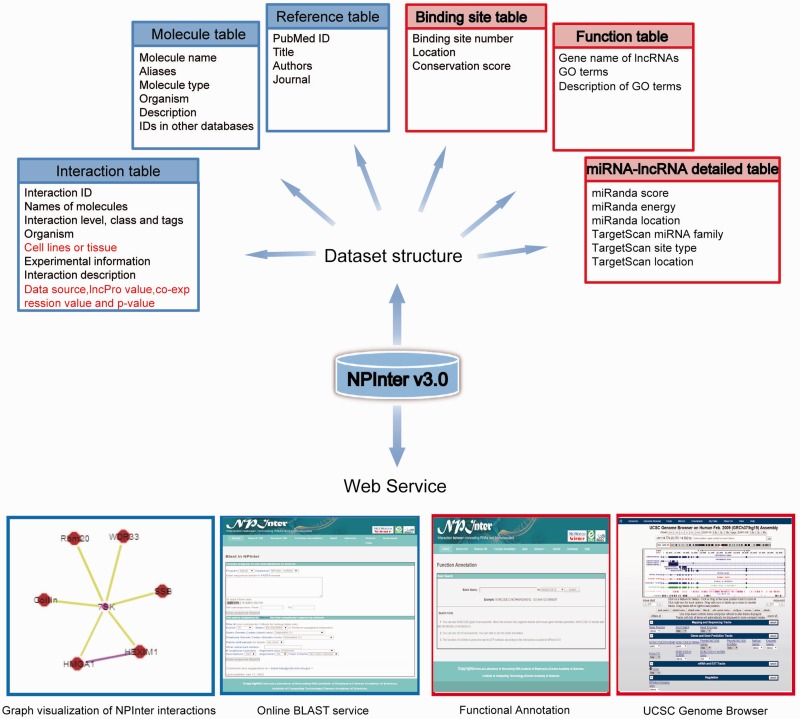
An overview of the NPInter v3.0 database. Improvements in this updated version are highlighted with a red border or by a red color. 191 × 183 mm (300 × 300 DPI).

An overview of NPInter v3.0 updates is shown in Figure 1.

## Data collection and annotation

**Figure 2:**
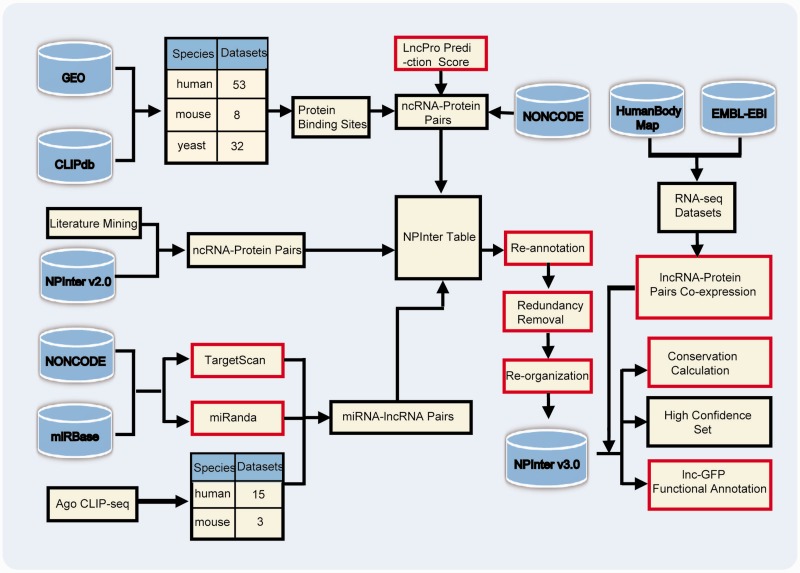
Workflow to collect interactions in NPInter v3.0. Red rectangles indicate that these steps need computational processing. Refer to main text for details. 169 × 123 mm (300 × 300 DPI).

The workflow of updating NPInter v3.0 is depicted in Figure 2. The interactions curated in NPInter v3.0 were mainly obtained from three different processing pipelines. We then re-annotated the molecules using specific IDs, removed redundant interactions and categorized interactions based on different standards. Meanwhile, we calculated gene co-expression scores between interacting molecules, and predicted lncRNAs’ functions. The detailed procedure is thoroughly explained in the following sections.

### Interactions curated from CLIP-seq data sets

In order to obtain all the *bona fide* interactions from high-throughput sequencing technologies, we collected all the available processed data from the CLIPdb database (33) and the Gene Expression Omnibus (GEO) (34) using keywords: RIP, CLIP, HITS-CLIP, PAR-CLIP and CLASH. After selection, we retained 111 associated datasets, including 18 Ago CLIP-seq datasets. We then converted the genomic coordinates to specific genome versions (hg19, mm9 and ce10) using the UCSC LiftOver Tool (35). Binding site locations were derived from the same article, same condition and same protein using the IntersectBed from BedTools (36). Then, we compared the union of the binding sites stored in BED format with the NONCODE v4.0 database, which is one of the most comprehensive reference databases of ncRNAs, and assigned NONCODE IDs to binding sites within ncRNAs. In addition, we provided a binding probability score per interaction calculated through LncPro (37) with default parameters, which effectively discriminated interacting and noninteracting lncRNA-protein pairs based on amino acid and nucleotide sequences. Furthermore, to assess the evolutionary conservation of each interaction-binding site, we first downloaded pre-computed sequence conservation scores (using the PhastCons (38) algorithm) across 46 vertebrate species for human, 30 vertebrate species for mouse, and 7 yeast species for yeast provided from the UCSC database, and then calculated an average PhastCons score (39) per binding site. The PhastCons program used a hidden Markov model-based method that estimated the probability that each nucleotide belonged to a conserved element, based on multiple alignments of selected species. The average PhastCons scores ranged from 0 to 1 where a value >0.1 showed some conservation between the species we considered, while a value >0.5 was considered highly conserved (40).

### miRNA–lncRNA interactions predicted by miRanda and TargetScan overlapped with Ago CLIP-seq datasets

ncRNAs from NONCODE v4.0 and miRNAs labeled as ‘broadly conserved’ or ‘conserved’ in TargetScan Release 7.0 (41) were acquired. AGO CLIP-seq datasets were retrieved from the GEO and CLIPdb databases. The conserved miRNA-target sites in ncRNAs were predicted using both TargetScan and miRanda (42) with the default parameters. The ncRNAs containing target sites that overlapped with any AGO CLIP cluster were considered as CLIP-supported interacting molecules. The resulting interactions were annotated as described. Moreover, the visualization of clusters in human and mouse were implemented in the Genome Browser and conservation scores were calculated as well.

### Interactions curated from literature mining and NPInter v2.0

In addition to data from the former version of the NPInter database, new datasets were obtained from the literature. First, we used a set of key words (listed in Supplementary Materials) to retrieve literature published since 12 April 2013 from PubMed and collected 991 articles. After selecting reports on new ncRNA-associated interactions, we extracted detailed information manually. Only interactions with strong support from experimental evidence were kept for further consideration.

### Re-annotation, redundancy removal, re-organization

After obtaining the interactions from the three main data sources mentioned earlier, we used NONCODE IDs or miRBase IDs to annotate ncRNAs and their interacting partners. Protein-related molecules were assigned with UniProt IDs, RefSeq IDs or UniGene IDs according to the type of molecules (proteins, protein-coding RNAs or DNA, respectively). Owing to the fact that the experiments and tissues (or cell lines) used to explore the interactions were varied and came from different papers using different descriptions, we manually uniformed the descriptions of tissues (or cell lines) and experiments. Next, the whole dataset was put through a filtration step to eliminate redundancy. Interactions with differences derived from various organisms, tissue or cell lines, or references were considered as independent records. As a final result, NPInter v3.0 provided a nonredundant and comprehensive resource of the ncRNA interactome. As different data sources had different confidences, we re-organized the interactions and defined 6 different data sources as described in Table 1. We added a data source per interaction in which different sources represented different probabilities describing a functional linkage between two molecules. In addition, we defined a high confidence set of interactions, which was obtained from literature mining and supported by low-throughput experiments, and represented the highest quality of interaction in the database. However, we did not sort data credibility according to the data sources, as the confidence of the data sources should be decided by the users, who should carefully compare them for any specific task at hand. In order to give guidance to users who want to balance different levels of accuracy, we provided three advices: First, the highest confidence data set was obtained from literature mining. Second, for the remaining five data sources listed in Table 1, the second was more confident than the third as the interactions obtained from the second data source were supported by high-throughput data and also supported by the predictive software. Third, the interactions which were predicted by miRanda and TargetScan combined with the Ago CLIP-seq data support were more reliable than the fifth and sixth terms. In summary, users can choose the most appropriate data based on their specific task at hand, according to the detailed description provided for each data source.

**Table 1:** The type of data sources in NPInter v3.0

**Data sources**	**Description**	**Number of interactions**
Literature mining	The interactions were obtained from literature mining.	8130
High-throughput data combined with LncPro prediction	The interactions were obtained from high-throughput data and were supported by LncPro prediction.	96 244
High-throughput data	The interactions were obtained from high-throughput data but were not supported by LncPro prediction.	252 317
miRanda and TargetScan with Ago CLIP data	The interactions were predicted by miRanda and TargetScan combined with Ago CLIP-seq data support.	33 366
miRanda with Ago CLIP data	The interactions were predicted by miRanda but were not predicted by TargetScan. The interactions were also supported by Ago CLIP-seq data.	39 447
TargetScan with Ago CLIP data	The interactions were predicted by TargetScan but were not predicted by miRanda. The interactions were also supported by Ago CLIP-seq data.	61 912

### Co-expression analysis of interactions

Gene co-expression values approximately evaluate co-regulation level and indicate the strength of the functional correlation between two genes (43, 44). We added gene expression correlation scores to the descriptions of interactions in order to help users to determine which interactions are more reliable. Higher co-expression scores in the interactions indicate that the interactions are more reliable if all the interactions were curated from the same data source. The co-expression scores in NPInter v3.0 were computed using an improved pipeline which will be described below, and made use of confident RNA-seq data from a single study to avoid batch effects. We downloaded the public RNA-seq data of human and mouse. The human data set was obtained from Human BodyMap 2.0 (ENA archive: ERP000546) across 16 tissues, while the mouse data set was retrieved from the EMBL-EBI database (ENA archive: ERP000591) across 6 different tissues. Next, we used Tophat (45) to map the reads to the reference genome (hg19 or mm9) and then calculated the FPKM of each reference gene using cuffnorm (46). The reference files for lncRNA genes and protein-coding genes were obtained from the NONCODE v4.0 and RefSeq databases, respectively. Utilizing the expression profiles of all reference genes, we then calculated the Pearson correlation coefficient of any two genes in each interaction curated in NPInter to represent the co-expression value. Finally, the co-expression values, as well as the p value, of each interaction were listed in the database. And one example was provided in the Supplementary Materials to illustrate the co-expression analysis.

### Functional annotation of lncRNAs

To enable researchers to have a better understanding of ncRNAs’ functions, we predicted the functions of lncRNAs through lnc-GFP (47) with the default parameters, a bi-colored network-based global function predictor according to the interactions curated in NPInter. The protein-protein interactions from the STRING v10 database (48) were integrated into the bi-colored network. A total of 8,710 lncRNA genes in NPInter v3.0 have been annotated with potential functions with a suitable parameter setting. Owing to the number of interactions, this version only supports human and mouse gene function prediction.

## Database content and structure

To date, the number of interactions in NPInter v3.0 increased to 491,416 in 22 species (Table 2) informed by 793 published articles, while the previous version of NPInter released in 2013 only contained 201,107 interactions from 18 species. Each updated interaction entry contains basic information including interaction ID, names of the two interacting molecules, interaction level, interaction class, tags, organism, tissue or cell type, experiment description, the interaction description, the data source and the co-expression value. In addition, the number of supporting CLIP reads is also provided for interactions from miRNA–lncRNA prediction with AGO CLIP-seq data, and the LncPro value is also provided for the interaction from high-throughput data. Levels were defined according to the types of interacting molecules such as ‘RNA–Protein’, ‘RNA–RNA’ and ‘RNA–DNA’. Tags of interactions were added according to the same definitions of NPInter v2.0. In order to improve usability to users, we added information on the relevant tissues or cell lines (Table 2), data source and co-expression values for each entry and assigned Ensembl IDs and RefSeq IDs to ncRNAs. For records with binding site information, every binding site is linked to the local UCSC Genome Browser, and an average PhastCons score is calculated.

**Table 2:** The statistics of interactions in different species and tissues (or cell lines) in NPInter v3.0

**Species**	**Number of interactions**	**Tissues or cell lines**	**Number of interactions**
*H. sapiens*	346 644	HEK293 cells	81 838
*M. musculus*	143 645	Mouse brain	63 461
*S. cerevisiae*	571	Human brain	33 165
*Agrobacterium tumefaciens*	208	HeLa cells	32 456
*Escherichia coli*	102	MDA-MB-231	33 168
*Caenorhabditis elegans*	65	Embryonic Stem Cell	26 471
*Drosophila melanogaster*	58	Cerebrums	22 062
*Kaposi sarcoma-*associated herpesvirus	41	CD4+ T cells	8928
Others	37	others	49 585

NPInter v3.0 consists of six major tables:

- Interaction table: the interaction table provided detailed information for entries. Take interaction ‘ncRI-3001387’ as an example: this interaction between HOTAIR and PCBP1 was discovered by RIP in gastric tissue of human with a description ‘A direct interaction between the HOTAIR and PCBP1 was confirmed through RNA immunoprecipitation coupled with quantitative real-time PCR’. Furthermore, this interaction was tagged as ‘ncRNA-Protein binding’ and divided into class ‘binding’ at a ‘RNA-protein; level. The interaction was curated from literature mining, and the co-expression value and *P*-value of this interaction from RNA-Seq data was −0.064 and 0.77, respectively.
- Molecule table: in this table, we described the name, aliases, molecule type, biotype, organism, a simple description and IDs from relevant databases for every molecule involved in interactions. In terms of ncRNAs, IDs in Ensembl, RefSeq, NONCODE v4.0 or miRBase are available while IDs in UniProt, UniGene and RefSeq are available for proteins or protein-related molecules.
- Reference table: the reference table listed general publication information and the MEDLINE standard article code (PMID) of literature recorded in the NPInter database.
- Binding site table: for interactions with binding sites generated from sequencing data, we provided the detailed position (chromosome, chromosome start and chromosome end) and PhastCons score for each site.
- Function table: in the function table, we described the lncRNA genes and their predicted functions in three columns, the gene name of the lncRNAs, GO terms and the description of the GO terms.
- miRNA–lncRNA interaction detailed information table: in this table, we provided the detailed information of interactions predicted from software coupled with AGO CLIP-seq data. For example, the interaction ‘ncRI-3356692’ is predicted by miRanda and TargetScan which is also supported by AGO CLIP reads. The number of supporting reads is 28, the interaction score from miRanda is 157, the interaction energy predicted by miRanda is −17.14 and the interaction region from miRanda is ‘chr1: 568999-569024’. In addition, the miRNA family from TargetScan was ‘let-7-5p/98-5p/miR-4458/4500’, the interaction type was ‘7mer-m8’ and the interaction region predicted by TargetScan was ‘chr1: 569017-569023’.

## Service update

The web interface of the NPInter v3.0 database has been re-designed and now provides a user-friendly web site to browse and search interactions. In addition, NPInter v3.0 incorporates Cytoscape for users to visualize interactions. An online BLAST service has been integrated as well, enabling users to search entries by sequence. Furthermore, local UCSC Genome Browser has been added in this update.

### Integration with a UCSC Genome Browser

As the UCSC Genome Browser (http://genome.ucsc.edu/) has been widely used, a local UCSC Genome Browser was built for *Homo sapiens*, *Mus musculus* and *Saccharomyces cerevisiae* in this new version of NPInter. The NPInter v3.0 track in the Genome Browser displays protein binding sites in these species. Associated tracks like NONCODE v4.0 lncRNA, NONCODE v4.0 lncRNA Gene, RefSeq Genes and Ensembl Genes are also shown in dense mode. Other general tracks, such as Conservation, are retrieved from the UCSC Genome Browser Database and users can change the display mode as they desire.

## Conclusion and future directions

Aiming to become a valuable and cutting edge resource for researchers who focus on exploring ncRNAs’ functions and molecular mechanisms, NPInter v3.0 has significantly increased the number of records and the amount of detailed information per interaction compared with the former version. NPInter v3.0 provides more detailed information about each individual entry: e.g. users can easily retrieve the basic information about an interaction, the data source where this interaction was curated from, the co-expression values, the exact interacting position, and tissue or cellular locations where this interaction occurred. In addition, in the new version, for records with binding site information, every binding site is linked to the local UCSC Genome Browser as well as an average PhastCons score. Moreover, we also assigned Ensembl IDs and RefSeq IDs to ncRNAs, in addition to the NONCODE ID. As for the interactions between lncRNAs and proteins, we obtained all the interactions supported by the high-throughput data in previous version. In NPInter v3.0, we not only curated the interactions supported by the new high-throughput data but also calculated an interaction score per interaction through LncPro software. The interactions whose scores are equal to or >50 are more reliable compared with the interactions whose scores are <50. In the previous version, we just obtained the interactions between miRNAs and lncRNAs from literature mining. In NPInter v3.0, we not only obtained the interactions from literature mining but also curated the interactions predicted by miRanda or TargetScan combined with Ago CLIP-seq data support. In addition, NPInter v3.0 not only updates the existing tools including BLAST and graph visualization of interactions to the new version, and also integrates Genome Browser services. Although there are some similar databases, NPInter v3.0 contains a more informative and organized data set with its own unique features. Take the RAID (49) and doRiNA (50) databases as examples: RAID only collects interactions in human and discards data generated by high-throughput techniques, while doRiNA mainly focuses on miRNAs without including other kinds of ncRNAs. NPInter contains interactions covering multiple species and ncRNAs, especially lncRNAs.

Furthermore, NPInter is a member of our platform for ncRNAs together with NONCODE, CNCI (51) and ncFANs (52). Consequently, NPInter v3.0 could present the most comprehensive transcriptome-wide map of interactions on ncRNAs for the scientific community.

As the amount of high-throughput sequencing data from a variety of species, tissues, cell lines and RNA- binding proteins increases, the authors will maintain and update the database. Importantly, although NPInter v3.0 has already improved the user interface and added new web services, we are planning to improve the performance of our computer servers, through expanding the memory and upgrading the processors, to provide a better user experience.

## Supplementary data

Supplementary data are available at *Database* Online.

Supplementary Data
