# Supplementary material for: NPInter v3.0: an upgraded database of noncoding RNA-associated interactions
Source: Database (Oxford). 2016 Apr 16;2016:baw057. doi: 10.1093/database/baw057 (PMC4834207; doi:10.1093/database/baw057)
Supplement: Supplementary Data [file supp_2016_baw057_index.html]

Supplementary Data 

# NPInter v3.0: an upgraded database of noncoding RNA-associated interactions

## Supplementary Data

files

- Supplementary Data - doc file
