# Supplementary material for: NPInter v3.0: an upgraded database of noncoding RNA-associated interactions
Source: Database (Oxford). 2016 Apr 16;2016:baw057. doi: 10.1093/database/baw057 (PMC4834207; doi:10.1093/database/baw057)
Supplement: Supplementary Data [file supp_baw057_supplementary.doc]

**Supplementary material**

Key words for searching:

ChIRP RNA, ChIRP Seq, circular RNA bind protein, CLIP bind, CLIP interaction, CLIP non coding RNA, CLIP RNA protein, CLIP Seq, CRAC bind, CRAC cross linking, CRAC RNA protein, HITS CLIP, iCLIP, lincRNA bind, lincRNA bind protein, lincRNA protein interaction, lncRNA bind, lncRNA bind protein, lncRNA protein interaction, ncRNA bind, ncRNA bind protein, ncRNA protein interaction, non coding RNA protein interaction, non coding RNA bind protein post transcriptional, noncoding RNA direct bind protein, PAR CLIP, RIP lincRNA, RIP lncRNA, RIP non coding RNA, RIP noncoding RNA, RIP Seq, RNA protein cross linking, RNA-RNA interaction, ChIRP, ChIRP MS

An example of the co-expression analysis.

In order to help users to understand the calculation pipeline of co-expression analysis better, we provided an example of the co-expression analysis. For the interaction “ncRI-3001982” we calculated the expression values of the molecules (“linc1287” and “Tip60”) in the interaction across 6 different tissues in mouse. The expression profile of Tip60 was “3.89, 0.21, 0.45, 9.65, 381.60, and 0.63” while the expression profile of linc1287 was “0.0017, 0.00, 0.02, 0.04, 0.23, and 0.05”. Then we calculated the Pearson correlation coefficient of the two genes using the “pearsonr" function in the “scipy.stats” package provided by the Python programming language. The Pearson correlation coefficient and p value of this interaction were 0.99 and 0.0001. The co-expression value and its corresponding p value demonstrated that linc1287 had an obvious expression correlation with Tip60, and further indicted that linc1287 and Tip60 had a high strength of functional correlation.
